# Hamiltonian transformability, fast adiabatic dynamics and hidden adiabaticity

**DOI:** 10.1038/s41598-021-84289-4

**Published:** 2021-02-25

**Authors:** Lian-Ao Wu, Dvira Segal

**Affiliations:** 1 Department of Theoretical Physics and History of Science, The Basque Country University (EHU/UPV), PO Box 644, 48080 Bilbao, Spain; 2 Ikerbasque, Basque Foundation for Science, 48011 Bilbao, Spain; 3 Chemical Physics Theory Group, Department of Chemistry, and Centre for Quantum Information and Quantum Control, University of Toronto, 80 Saint George St., Toronto, ON M5S 3H6 Canada; 4 Department of Physics, University of Toronto, Toronto, ON M5S 1A7 Canada

**Keywords:** Information theory and computation, Quantum information, Quantum mechanics, Quantum simulation, Qubits

## Abstract

We prove the existence of a unitary transformation that enables two arbitrarily given Hamiltonians in the same Hilbert space to be transformed into one another. The result is straightforward yet, for example, it lays the foundation to implementing or mimicking dynamics with the most controllable Hamiltonian. As a promising application, this existence theorem allows for a rapidly evolving realization of adiabatic quantum computation by transforming a Hamiltonian where dynamics is in the adiabatic regime into a rapidly evolving one. We illustrate the theorem with examples.

## Introduction

Understanding quantum dynamics and control is essential to modern quantum technologies such as adiabatic quantum computation^1,2^. A quantum dynamical processes is driven by its corresponding Hamiltonian, where the Hamiltonian represents a physical realization. For instance, spin dynamics can be driven by the Zeeman Hamiltonian, which is physically realized by applying magnetic fields^3^. Different realized dynamics, for example fast vs. adiabatically controlled passage^4–6^ may seem remote from one another. However, in this paper we show that they can well be intimately related.

For example, a physical realization of adiabatic quantum computation (AQC) suffers from its slowness, with the resultant destructive effects of decoherence and the occurrence of quantum phase transitions during dynamics^7–10^. Here, we prove rigorously that different dynamics, described by two Hamiltonians defined on the same Hilbert space, can always be transformed into one another. As a consequence, the physical outcome of AQC can be made equivalent to the outcome of a dynamical process that can be extremely fast. This relationship between different dynamics is based on a straightforward but profound proposition described below, implying, for example, that an adiabatic process may be physically realized with a fast Hamiltonian. Similarly, it implies a hidden adiabaticity amongst rapid dynamics.

## The transformability proposition

Given any two Hamiltonians, ${\hat{H}}$ and ${\hat{h}}$ in the same Hilbert space, which can be time-independent or time-dependent, the corresponding Schrödinger equations are

1 $$\begin{aligned} i\partial _t{\hat{U}}={\hat{H}}(t){\hat{U}}, \end{aligned}$$

and

2 $$\begin{aligned} i\partial _t{\hat{u}}={\hat{h}}(t){\hat{u}}, \end{aligned}$$

where ${\hat{U}}$ and ${\hat{u}}$ are propagators of ${\hat{H}}(t)$ and ${\hat{h}}(t)$, respectively.

### Proposition

*Two Hamiltonians* ${\hat{H}}$ *and* ${\hat{h}}$ *can always be transformed into one another.* *Mathematically, this claim can be expressed as: For given* ${\hat{H}}$ and ${\hat{h}}$, *there exists at least one unitary operator* ${\hat{S}}$ *such that*

3 $$\begin{aligned} {\hat{h}}={\hat{S}}^{\dagger } {\hat{H}} {\hat{S}}-i{\hat{S}}^{\dagger }\dot{{\hat{S}}}, \end{aligned}$$

*and*

4 $$\begin{aligned} {\hat{H}}={\hat{S}} {\hat{h}} {\hat{S}}^{\dagger }-i{\hat{S}}\dot{{\hat{S}}}^{\dagger }, \end{aligned}$$

*where the overdot indicates a time derivative*.

### *Proof*

The operator ${\hat{S}}$ enables the transformation ${\hat{U}}={\hat{S}}{\hat{u}}$. Substituting it into the Schrödinger equation (1), we obtain Eq. (2) with the *effective* Hamiltonian ${\hat{h}}={\hat{S}}^{\dagger } {\hat{H}} {\hat{S}}-i{\hat{S}}^{\dagger }\dot{{\hat{S}}}$. Similarly, if we begin with the Schrödinger equation (2) we transform it to Eq. (1) by identifying its Hamiltonian with ${\hat{H}}={\hat{S}} {\hat{h}} {\hat{S}}^{\dagger }-i{\hat{S}}\dot{{\hat{S}}}^{\dagger }$. Because the solutions ${\hat{u}}$ and ${\hat{U}}$ of the Schrödinger equations (1) and (2) always exist, so does the product ${\hat{U}}{\hat{u}}^{\dagger }$. By setting ${\hat{S}}={\hat{U}}{\hat{u}}^{\dagger }$, we can reproduce the Hamiltonians (3) and (4) and therefore formally prove the universal existence of the unitary transformation ${\hat{S}}$. In other words, there is *always* a unitary transformation that enables two arbitrarily given Hamiltonians in the same Hilbert space to be transformed into one another. We term this property transformability, and the two Hamiltonians ${\hat{H}}$ and ${\hat{h}}$ are *transformable*. The special case of the proposition with ${\hat{h}}$ being time-independent was proven a quarter-century ago in Ref.^11^. In this context, Ref.^12^ proved a universal existence theorem of constant Hamiltonians for shortcuts to adiabaticity (STA)^7^. The theorem states that time-independent shortcuts can be immediately derived from the generalized phase mimicking the adiabatic evolution. This provides a direct way to implement a constant shortcut, an application that goes beyond the proof of existence that we provide here.

Note that in the STA protocol, corresponding to the adiabatic Hamiltonian (${\hat{h}}$), a shortcut to the adiabatic Hamiltonian (${\hat{H}}$) is identified with a counter-diabatic term^7^. Based on our theorem, a unitary ${\hat{S}}(t)$ is guaranteed to exist, allowing the transformation between the adiabatic and the STA Hamiltonians according to Eqs. (3)–(4) , which was exemplified e.g. in Ref.^13^ for the Rabi model.

## Rapid adiabatic quantum computation

Adiabatic quantum computation is one of the most promising candidates to realize quantum computing^14^. The approach is based on the adiabatic theorem: The solution to a computational problem of interest is encoded in the ground state of a potentially complicated Hamiltonian. To approach the solution, one prepares a system with a simpler Hamiltonian and initializes it at its ground state. By evolving the Hamiltonian sufficiently slowly towards the desired (complex) one, the adiabatic theorem guarantees that the system follows the instantaneous ground state, finally realizing the target ground state. Evidently, the slowness of AQC could be the main impediment to its utility for quantum algorithms.

The universal transformability property suggests that a slow AQC process ${\hat{u}}$ can be mapped onto a fast quantum process ${\hat{U}}$—that is more controllable, and suffers reduced decoherence during processing. Previous studies on adiabatic processes have shed light on the transformability in case studies. For example, Ref.^15^ examined conditions for adiabaticity by transforming a Hamiltonian in a specific non-adiabatic regime (for instance, due to the resonance phenomenon) to another Hamiltonian in the adiabatic regime. Our proposition here points to the mathematical proof of the universal *existence* of (at least one) unitary operator ${\hat{S}}(t)$ connecting an arbitrarily-given (could be adiabatic) Hamiltonian and another (adiabatic or not) arbitrarily-given Hamiltonian. Note that as a convention, we use lower (upper) cases to denote the slow (fast) dynamics throughout the paper. Consequently, AQC can be physically realized by a fast process. Nevertheless, if *“fastness”* means less number of quantum gates, it might contradict results of computational complexity theory. In this case, the total number of gates of the quantum process described by ${\hat{U}}$ should be compensated in the implementation of ${\hat{S}}$.

The eigenstate $|E(T)\rangle$ of the problem Hamiltonian at time *T* is given by implementing the adiabatic process

5 $$\begin{aligned} |E(T)\rangle \sim {\hat{u}}|E(0)\rangle ={\hat{S}}^{\dagger } (T) {\hat{U}} (T) |E(0)\rangle . \end{aligned}$$

The second equality suggests to physically implement $|E(T)\rangle$ by the following circuit: the first gate ${\hat{U}} (T)$ is governed by ${\hat{H}}$. The transformation ${\hat{S}}^{\dagger } (T)$ acts on the output.

## Adiabatic algorithms and their fast counterparts

Consider now the proposition in the context of a realistic AQC, i.e. an ensemble of qubits described by a family of slowly-varying Hamiltonians,

6 $$\begin{aligned} {\hat{h}}=\Gamma (t) \sum _{i}{\hat{X}}_{i}+{\hat{h}}_P(\{{\hat{Z}}_i\}). \end{aligned}$$

Here, $\Gamma (t)$ is large at $t=0$, and slowly evolves towards zero at $t=T$. The Hamiltonian ${\hat{h}}_P(\{{\hat{Z}}_i\})$ contains the ${\hat{Z}}_i$ component of the *i*-th qubit. The solution of a *hard* problem is encoded within ${\hat{h}}_P$. For example, Grover’s search problem^14^ is realized with

7 $$\begin{aligned} {\hat{h}}_P({{\hat{Z}}_i})={\hat{I}}-|B\rangle \langle B|, \end{aligned}$$

where $|B\rangle$ is the *marked* state, and $|B\rangle \langle B|$ is a function of ${\hat{Z}}_i$.

Alternatively, in the D-Wave system, the Hamiltonian (6) is given by

8 $$\begin{aligned} {\hat{h}}_P({{\hat{Z}}_i})=\sum _{i} h_{i}{\hat{Z}}_{i}+\sum _{ij} J_{ij}{\hat{Z}}_i {\hat{Z}}_j, \end{aligned}$$

with the parameters $h_i$ and $J_{ij}$. Applying a fast magnetic field, we can enable the corresponding fast-varying Hamiltonian

9 $$\begin{aligned} {\hat{H}}=\gamma (t)\sum _{i}{\hat{X}}_{i}+{\hat{h}}_P(\{e^{-i\phi _{i}(t) {\hat{X}}_{i}}{\hat{Z}}_{i}e^{i\phi _{i}(t){\hat{X}}_{i}}\}), \end{aligned}$$

where $\gamma (t)=\Gamma (t)+{\dot{\phi }}$ is a fast-varying function. Here, the transformation matrix is given by ${\hat{S}}(t)=\Pi _i e^{-i\phi _i(t){\hat{X}}_i }$. Thus, instead of evolving the system slowly under ${\hat{h}}$, the two gates ${\hat{U}}(t)$ and ${\hat{S}}(t)$ should be realized, Eq. (5), allowing for a fast implementation.

## Built-in adiabaticity

The discussion above focuses on implementing fast dynamics to achieve the adiabatic result, i.e. to replace slow dynamics by fast dynamics. Here we show that the opposite is also the case, That is, fast dynamics can be shown to have a “hidden adiabaticity”. As an example, consider a qubit under external fields, with the NMR-type Hamiltonian

10 $$\begin{aligned} {\hat{H}}=\frac{\omega _0(t)}{2}{\hat{Z}}+g\left[ {\hat{X}}\cos \phi (t) +{\hat{Y}}\sin \phi (t)\right] . \end{aligned}$$

Here, ${\hat{X}}$, ${\hat{Y}}$ and ${\hat{Z}}$ are the Pauli operators, $\omega _0(t)$ and $\phi (t)$ potentially depend on time and are allowed to be fast-varying. A unitary transformation ${\hat{S}}=\exp \left[ i\frac{\theta (t)-\phi (t)}{2}{\hat{Z}}\right]$ brings ${\hat{H}}$ into ${\hat{h}}$,

11 $$\begin{aligned} {\hat{h}}=\frac{\omega _0(t)+{\dot{\theta }}-{\dot{\phi }}}{2}{\hat{Z}} +g\left[ {\hat{X}}\cos \theta (t)+{\hat{Y}}\sin \theta (t)\right] . \end{aligned}$$

We assume that *g* is a constant and that the newly introduced time-dependent parameter $\theta (t)$, as well as $\frac{\omega _0(t)+{\dot{\theta }}-{\dot{\phi }}}{2}$ are controlled such that they vary slowly. The transformation ${\hat{S}}$ thus brings the system into the adiabatic domain. In other words, a system driven by fast-varying ${\hat{H}}$ has built-in hidden adiabaticity characterized by ${\hat{h}}$.

In the particular case where $\phi (t)=\omega t$ and $\omega _0$, $\omega$ are constants, we can easily obtain the solution, that is the time evolution operator corresponding to ${\hat{H}}$,

12 $$\begin{aligned} {\hat{U}}=\exp \left( -i\frac{\omega {\hat{Z}}}{2} t\right) \exp \left( -i\frac{2g{\hat{X}}-\Omega {\hat{Z}}}{2}t\right) , \end{aligned}$$

where $\Omega =\omega -\omega _0$. We can control parameters and realize the function $\theta (t)=\Omega t$, resulting in

13 $$\begin{aligned} {\hat{u}}=\exp \left( -i\frac{\Omega {\hat{Z}}}{2}t\right) \exp \left( -i\frac{2g{\hat{X}}-\Omega {\hat{Z}}}{2}t\right) . \end{aligned}$$

The instantaneous eigenstates of ${\hat{h}}=g\exp \left( -i\frac{\Omega {\hat{Z}}}{2}t\right) {\hat{X}}\exp \left( i\frac{\Omega {\hat{Z}}}{2}t\right)$ are

14 $$\begin{aligned} |E_{\pm }(t)\rangle =\exp \left( -i\frac{\Omega {\hat{Z}}}{2} t \right) |\pm \rangle . \end{aligned}$$

These states are proportional to the wave function ${\hat{u}}|\pm \rangle$ (${\hat{X}}|\pm \rangle =\pm |\pm \rangle$) as stated by the adiabatic theorem for the adiabatic regime $g\gg \Omega$. Therefore, in order to physically realize $|E_{\pm }(T)\rangle$, say at $T=\pi /2\Omega$ when ${\hat{h}}(T)=g{\hat{Y}}$, one needs to implement two gates: ${\hat{U}}(T)$, then ${\hat{S}}^\dagger (T)= \exp (i\frac{\pi \omega _0}{4\Omega } {\hat{Z}})$.

We now come to a simple but nontrivial corollary following immediately from the Transformability proposition.

## The transformability corollary at different times

Let ${\hat{H}}$ (the fast Hamiltonian) be a function of the normalized or scaling time $\tau =t/T$, where *T* a characteristic time of the dynamical system. Equation (1) can then be rewritten as

15 $$\begin{aligned} i\partial _\tau {\hat{U}}(\tau )=T{\hat{H}}(\tau ){\hat{U}}(\tau ). \end{aligned}$$

Likewise,

16 $$\begin{aligned} i\partial _\tau {\hat{u}}(\tau )=T'{\hat{h}}(\tau ){\hat{u}}(\tau ), \end{aligned}$$

where $\tau =t'/T'$ and the latter describes a slower process so that $T<T'$, $t'(T')$ is the real time (characteristic time) of the Schrödinger equation (16). The scaling times of two equations may be identical or different. Here we set the same scaling time $\tau$ with the constraint $t'/T'=t/T$. As proved in the transformability proposition, mathematically there is at least one unitary operator ${\hat{S}}$ such that

17 $$\begin{aligned} T'{\hat{h}}={\hat{S}}^{\dagger } T{\hat{H}} {\hat{S}}-i{\hat{S}}^{\dagger }\partial _\tau {{\hat{S}}}, \end{aligned}$$

and

18 $$\begin{aligned} T{\hat{H}}={\hat{S}} T'{\hat{h}} {\hat{S}}^{\dagger }-i{\hat{S}}\partial _\tau {{\hat{S}}}^{\dagger }, \end{aligned}$$

for a given scaling time $\tau$. The simplest non-trivial example is ${\hat{S}}=1$, such that ${\hat{H}}=\frac{T'}{T}{\hat{h}}$ and ${\hat{U}}(\tau )={\hat{u}}(\tau )$. The latter equality, rewritten as ${\hat{U}}(t)={\hat{u}}(\frac{T'}{T}t)$ with $T<T'$, is an exact proof that the runtime of an adiabatic quantum process can be reduced $\frac{T'}{T}$ times—exact trade-off between energy and time. Specifically, Eq. (12) can be rewritten as

19 $$\begin{aligned} {\hat{U}}(\tau )={\hat{u}}(\tau )=\exp (-i\pi Z\tau )\exp \left( -i(T g{\hat{X}}-\pi {\hat{Z}})\tau \right) , \end{aligned}$$

where $\tau =t/T=t'/T'$, $gT=g'T'$ and we have set $\omega _0=0$. ${\hat{U}}(\tau )$ (${\hat{u}}(\tau )$) may denote a fast (adiabatic) evolution if $T'$ is in the adiabatic regime while *T* is not in. The one-qubit gate ${\hat{U}}$ takes shorter total time $t=T$ (setting $\tau =1$) with strength *g*. Meanwhile, the gate ${\hat{u}}$ takes longer time $t'=T'$ but with a reduced strength $g'=gT/T'$. This reflects that the trade-off between energy and time may cause the fastness of evolution for this specific case. This result suggests a strategy of experimentally implementing an expedited adiabatic processes: simply enhancing the strength of the driving Hamiltonian to its strongest possible value.

In general, the universal existence of *S* and the equality

20 $$\begin{aligned} {\hat{u}}\left( \frac{t'}{T'}\right) ={\hat{S}}^{\dagger }\left( \frac{t}{T}\right) {\hat{U}}\left( \frac{t}{T}\right) \end{aligned}$$

manifests that an adiabatic quantum algorithm can always be mimicked by at most two fast gates where $T' \gg T$ is in the adiabatic regime.

## Conclusion

Two arbitrarily given Hamiltonians within the same Hilbert space can be always transformed to each other via a unitary transformation. This seemingly simple but rigorous theorem is powerful: It allows one to implement a slowly varying evolution within a fast protocol, which is less susceptible to errors. We exemplified this result on a qubit system and on problems in the context of quantum adiabatic computing. The transformability of open quantum system Hamiltonians is left for future work.
